# Prevalence of electronegative electroretinograms in a healthy adult cohort

**DOI:** 10.1136/bmjophth-2021-000751

**Published:** 2021-07-19

**Authors:** Xiaofan Jiang, Taha Bhatti, Ambreen Tariq, Katie M Williams, Isabelle Chow, Talib Dar, Andrew R Webster, Pirro G Hysi, Christopher J Hammond, Omar A Mahroo

**Affiliations:** 1 Institute of Ophthalmology, University College London, London, UK; 2 Section of Academic Ophthalmology, School of Life Course Sciences, FoLSM, Kings College London, London, UK; 3 Department of Twin Research and Genetic Epidemiology, King’s College London, London, UK; 4 NIHR Biomedical Research Centre, Moorfields Eye Hospital NHS Foundation Trust and the UCL Institute of Ophthalmology, London, UK; 5 Physiology, University of Cambridge, Cambridge, UK

**Keywords:** retina, electrophysiology

## Abstract

**Objective:**

An electronegative electroretinogram (ERG) can indicate important ocular or systemic disease. This study explored the prevalence of electronegative responses to dark-adapted stimuli in a largely healthy cohort.

**Methods and Analysis:**

211 participants recruited from the TwinsUK cohort underwent ERG testing incorporating international standard (International Society for Clinical Electrophysiology of Vision (ISCEV)) protocols and additional stimuli. Responses were recorded using conductive fibre electrodes, following pupil dilation and 20 min dark adaptation. Responses analysed were to the ISCEV standard and strong flashes (3.0 and 10 cd/m^2^ s), and to additional white flashes (0.67–67 cd/m^2^ s). A-wave and b-wave amplitudes were extracted; b:a ratios were calculated and proportions of eyes with ratios<1 were noted.

**Results:**

Mean (SD) age was 62.4 (11.4) years (median, 64.3; range 23–86 years). 93% were female. Mean (SD) b:a ratios for right and left eyes, respectively, were 1.86 (0.33) and 1.81 (0.29) for the standard flash, and 1.62 (0.25) and 1.58 (0.23) for the stronger flash; average b:a ratio was lower for the stronger flash (p<0.0001). No waveforms were electronegative. For additional flashes, b:a ratio decreased with increasing flash strength. No electronegative waveforms were seen except in three eyes (0.7%) for the strongest flash; in some cases, drift in the waveform may have artefactually reduced the b:a ratio.

**Conclusion:**

For standard dark-adapted stimuli, no participants had electronegative waveforms. The findings support the notion that electronegative waveforms (in response to standard flash strengths) are unusual, and should prompt further investigation.

**Key messages**

**What is already known about this subject?**

- Electronegative electroretinogram (ERG) responses to standard dark-adapted stimuli are associated with a number of congenital and acquired conditions, the latter including paraneoplastic retinopathies.

**What are the new findings?**

- This study investigated whether electronegative ERGs might be observed in some healthy subjects: in a large healthy adult cohort, no participant exhibited an electronegative ERG in response to standard stimuli.

**How might these results change the focus of research or clinical practice?**

- These findings support the notion that electronegative ERGs to standard dark-adapted stimuli are an unusual finding and should prompt further clinical history and/or investigation.

## Introduction

**Figure 1:**
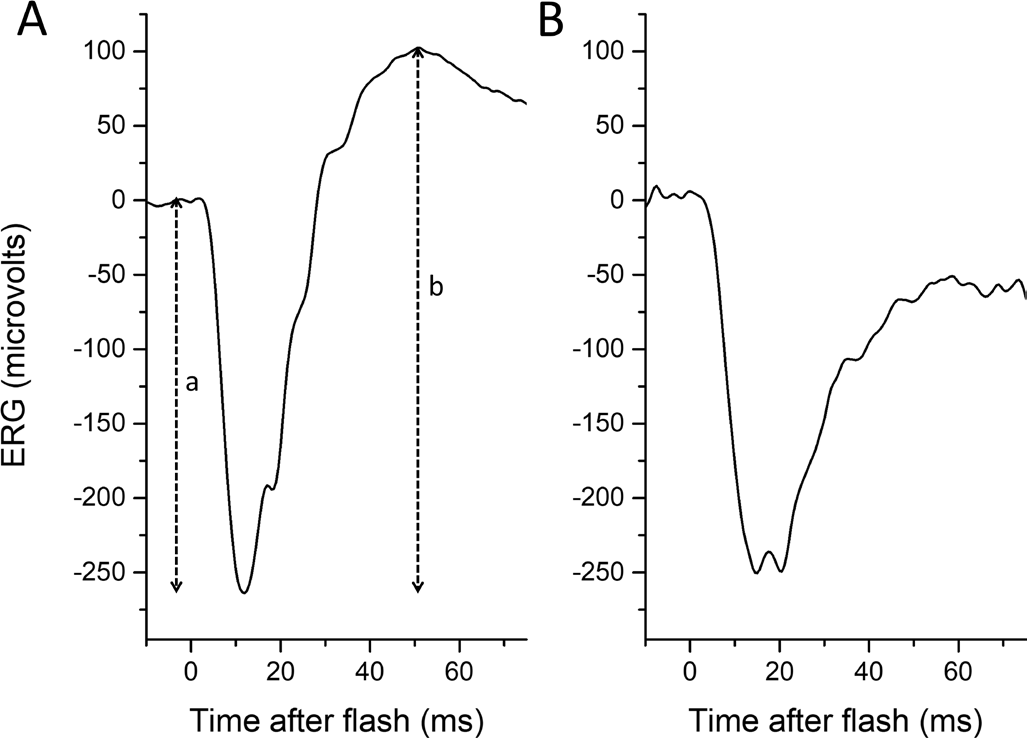
Normal (left panel) and electronegative (right panel) dark-adapted electroretinograms (ERGs) to a standard 10 cd/m^2^ s white flash. (A) Response from healthy adult. The a-wave amplitude is measured from baseline to the trough of the a-wave (depicted by dashed vertical line labelled “*a*”), and the b-wave amplitude is measured from the trough of the a-wave to the peak of the b-wave (dashed vertical line labelled “*b*”). (B) Response from the symptomatic eye of a patient with acquired unilateral nyctalopia, thought to be of autoimmune aetiology. In the patient’s recording, the b-wave amplitude is smaller than that of the a-wave, and the waveform is termed electronegative.

The electroretinogram (ERG) response to a full-field flash stimulus is biphasic, consisting of an initial, negative-going waveform (the a-wave, arising from hyperpolarising currents in photoreceptors and OFF bipolar cells), followed by a positive-going waveform (the b-wave, shaped largely by currents in ON and OFF bipolar cells.) In the dark-adapted state, the a-wave arises largely from rod photoreceptors (the hyperpolarisation results from phototransduction, which shuts off a depolarising current flowing into the photoreceptor outer segment) and the b-wave from rod-driven ON bipolar cells, although the cone system also contributes to both components. A-wave amplitudes are measured from baseline to a-wave trough, and b-wave amplitudes from a-wave trough to b-wave peak. If the b-wave amplitude is less than that of the a-wave (b:a ratio less than 1), the waveform is termed electronegative (figure 1), with the term usually being applied when the a-wave is of normal (or near-normal) amplitude.^1^

In response to standard dark-adapted flashes (as defined by the International Society for Clinical Electrophysiology of Vision (ISCEV)),^1 2^ an electronegative ERG usually indicates dysfunction occurring after phototransduction (eg, at the level of the photoreceptor synapse or bipolar cell). The finding of an electronegative ERG, particularly a normal-sized a-wave and reduced b-wave, is of clinical significance,^3–5^ and can narrow the differential diagnosis considerably (table 1). In clinical patient cohorts undergoing electroretinography, proportions with electronegative ERGs range between approximately 2.9% and 6.6%.^6–9^ In the context of acquired disease, an electronegative ERG can indicate an inflammatory or paraneoplastic process, with a particular pattern found in melanoma-associated retinopathy, explicable by the presence of circulating autoantibodies to the TRPM1 channel expressed by retinal ON bipolar cells.^10 11^ If the a-wave is additionally reduced, this indicates impairment at the level of photoreceptor outer segments; in such cases, an electronegative waveform might not always indicate inner retinal pathology.

**Table 1:** Some causes of electronegative electroretinogram waveforms

Inherited	X-linked retinoschisis Congenital stationary night blindness Batten disease Duchenne muscular dystrophy Other genetic conditions		
Acquired	Vascular	Central retinal artery/vein occlusion	
	Toxic	Vigabatrin Quinine Methanol Siderosis	
	Inflammatory or autoimmune	Paraneoplastic	Melanoma-associated retinopathy Carcinoma-associated retinopathy
		Non-paraneoplastic	Birdshot uveitis Other inflammatory/autoimmune

In the authors’ experience, electronegative ERGs can sometimes be unexpected, and do not appear to fit initially with the clinical picture, raising the question whether a minority of healthy subjects might display an electronegative waveform that does not indicate clinical pathology. Reference datasets do not include electronegative waveforms, but this could be confounded by the fact that such waveforms might be retrospectively excluded, as it cannot be certain that the individual did not have undiagnosed retinal pathology. Prospective recordings from a large cohort of healthy subjects are helpful to explore this question.

In this study, we investigated whether electronegative waveforms might be observed in some healthy subjects. We analysed ERG waveforms from a largely healthy sample of over 200 adults (aged between 23 and 86 years) in response to ISCEV standard protocols, and additional stimuli. These responses had been recorded prospectively in the TwinsUK cohort for an investigation of heritability of retinal response parameters,^12^ and no recordings were excluded on the basis of electronegativity. In the present study, we looked at the distribution of b:a ratios, looking specifically for the presence of electronegative ERGs in the dark-adapted responses.

## Materials and methods

Adult participants were recruited from the TwinsUK cohort,^12^ which is a registry of largely healthy adult twins, who have volunteered to participate in research studies at St Thomas’ Hospital, London.^13^ Participants underwent full-field ERG testing which incorporated the ISCEV standard protocol.^1 2^ Participants gave informed consent; the study had local research ethics committee approval, and complied with the tenets of the Declaration of Helsinki. TwinsUK takes account of feedback from participants; patients and public were not involved specifically in the design of the present study.

Pupils were pharmacologically dilated and participants underwent 20 min dark adaptation prior to commencement of stimuli. The stimuli delivered were the standard ISCEV dark-adapted stimuli (flashes delivering 0.01, 3.0 and 10 photopic cd/m^2^ s, conventionally termed the DA 0.01, DA 3 and DA 10), followed by additional white flashes (delivering 0.67, 4.0, 13 and 67 photopic cd/m^2^ s). The ISCEV standard stimuli were from LEDs, while the additional flashes were from a xenon flash gun. The additional stimuli were delivered as part of an ongoing study aiming to explore application of mathematical models to a-wave kinetics, and in order to minimise flash duration, xenon flash, rather than LED, stimuli were used. The corresponding flash strengths in scotopic units were 0.03, 7.5 and 25 scotopic cd/m^2^ s for the ISCEV stimuli (DA 0.01, 3 and 10 flashes, respectively), and were 1.0, 6.2, 21 and 104 scotopic cd/m^2^ s for the additional xenon flashes. The interstimulus interval ranged from 5 s for the weaker stimuli to 20 s for the stronger flashes. The flash strengths given above in photopic units were as measured independently using a photometer with photopic filter (and confirmed by a subsequent calibration by the manufacturer). The strengths in scotopic units were as given by the Espion software for each stimulus.

ERGs were recorded using a conductive fibre electrode placed in the inferior conjunctival fornix (with consistency of position checked during and after recordings as this can affect amplitudes).^14^ Recordings were made simultaneously from both eyes. Indifferent skin electrodes were placed on the temples, and a ground electrode on the forehead. Stimuli were delivered using the Diagnosys ColorDome running Espion software (Diagnosys, Lowell, Massachusetts, USA). Filter settings for recordings were as set by the manufacturer (high pass 0.312 Hz, low pass 300 Hz). For the standard ISCEV stimuli analysed in this study, the Espion ‘auto-reject’ function and the prespecified ‘drift removal’ functions were not enabled, but the operator had the opportunity to manually reject a response (using criteria specified below) prior to conclusion of recordings. For the xenon flash stimuli, the ‘auto-reject’ and manual rejection functions and ‘drift removal’ were not enabled; exclusion of artefactual traces was performed after conclusion of recordings.

Responses elicited by the dark-adapted ISCEV standard flash (3.0 photopic cd/m^2^ s) and strong flash (10.0 photopic cd/m^2^ s) stimuli were analysed, as well as those elicited by the additional white flashes. Very noisy responses can distort the average, and so were excluded using criteria similar to those previously described.^12 15^ The response was viewed over the full acquisition time window (ranging from 20 ms prior to flash delivery to 100 ms post flash). Those responses in which there was significant blink artefact (defined as a sharp, large amplitude deflection in the trace not consistent with a retinal response) or clear drift (sustained upward or downward deflection of more than 20 microvolts over the prestimulus window) were excluded, as well as those in which there was significant mains electrical interference (50 Hz). Typically, fewer than 10% of the responses were removed (in many cases, none were removed). Responses were averaged, typically from 4 to 6 flash presentations (or from up to 20 flash presentations for the lowest strength xenon flash). Where the averaged response showed some shallow baseline drift, the Espion postacquisition ‘trend removal’ function was used. Importantly, the presence of an electronegative waveform was not used as a criterion for trace rejection. B-wave and a-wave amplitudes were extracted for each subject and b:a amplitude ratios calculated. A test of normality (Shapiro-Wilk) was applied, and parametric or non-parametric tests chosen for subsequent comparisons based on whether the data were consistent or inconsistent with a normal distribution.

## Results

### Cohort demographics

Dark-adapted ERGs were analysed from 211 participants (422 eyes). For one participant, the stronger flash was not delivered. Mean (SD) age was 62.4 (11.4) years. The median age was 64.3 years (range 23–86). The majority (93%) were female, and 97% were of white European ancestry, reflecting the demographics of the TwinsUK cohort. The majority (>90%) did not report any eye condition expected to affect the full-field ERG. Eighteen individuals were noted to have the following retinal conditions: age-related macular degeneration (four participants), diabetes (five participants), previous retinal detachment (two participants), unspeciﬁed retinal problems (two participants), glaucoma (three participants) and glaucoma suspect (two participants). Axial lengths obtained by optical biometry (and averaged for the two eyes) were available for 76% of participants. Mean (SD) axial length was 23.36 (1.18) mm. The median was 23.28 mm (range from 20.06 to 27.12 mm). No participants were excluded on the basis of axial length.

### ISCEV standard dark-adapted flashes (DA 3 and DA 10)

Table 2 summarises the mean, SD, minimum and maximum values for each parameter. For the DA 3 flash, mean (median, SD) a-wave and b-wave amplitudes were, respectively, 145.2 (140.7, 37.9) and 264.0 (253.0, 62.8) microvolts for right eyes, and 147.3 (145.1, 32.8) and 263.9 (256.6, 58.9) microvolts for left eyes. For the stronger (DA 10) flash, respective values were 171.6 (167.3, 42.0) and 273.8 (262.6, 62.0) microvolts for right eyes, and 174.6 (170.9, 36.0) and 274.4 (268.3, 58.7) microvolts for left eyes.

**Table 2:** Average, SD and range of parameter values to standard and additional stimuli (b:a ratios given in italics)

Stimulus type	Stimulus strength (cd/m^2^ s)	Parameter	Right eye							Left eye						
			Mean	SD	Median	Min	Max	2.5th percentile	97.5th percentile	Mean	SD	Median	Min	Max	2.5th percentile	97.5th percentile
Standard dark-adapted flashes (LED)	3.0	a-wave (µV)	145.23	37.91	140.70	66.81	274.70	81.75	229.90	147.28	32.84	145.10	74.44	239.30	96.08	221.40
		b-wave (µV)	264.03	62.81	253.00	150.90	490.60	165.10	404.10	263.89	58.89	256.60	141.90	478.60	172.00	397.90
		*b:a ratio*	*1.86*	*0.33*	*1.81*	*1.12*	*3.25*	*1.33*	*2.64*	*1.81*	*0.29*	*1.79*	*1.13*	*2.81*	*1.30*	*2.52*
	10	a-wave (µV)	171.58	41.96	167.25	88.46	318.70	100.80	263.90	174.61	36.02	170.85	93.32	278.30	117.20	258.40
		b-wave (µV)	273.82	61.98	262.60	149.90	489.60	178.90	405.60	274.44	58.68	268.25	149.70	498.80	182.30	404.70
		*b:a ratio*	*1.62*	*0.25*	*1.60*	*1.15*	*2.66*	*1.20*	*2.30*	*1.58*	*0.23*	*1.57*	*1.04*	*2.36*	*1.19*	*2.10*
Additional xenon flashes	0.67	a-wave (µV)	48.06	19.43	44.37	13.36	118.39	19.42	90.05	48.87	17.87	45.51	15.36	112.20	22.33	94.47
		b-wave (µV)	202.71	50.12	198.68	106.41	401.41	120.61	316.70	203.56	48.64	201.60	99.66	364.72	122.06	307.47
		*b:a ratio*	*4.66*	*1.61*	*4.26*	*2.21*	*13.15*	*2.72*	*8.85*	*4.50*	*1.37*	*4.24*	*2.37*	*11.69*	*2.54*	*7.47*
	4.0	a-wave (µV)	129.04	36.87	124.85	32.96	234.10	71.86	214.21	130.48	33.41	126.81	29.89	227.00	76.66	199.87
		b-wave (µV)	251.40	59.04	245.63	122.87	465.41	157.74	363.89	250.55	55.39	248.36	127.96	409.48	158.96	373.67
		*b:a ratio*	*2.03*	*0.50*	*1.96*	*1.22*	*6.25*	*1.32*	*3.20*	*1.98*	*0.50*	*1.95*	*1.16*	*6.93*	*1.40*	*3.01*
	13	a-wave (µV)	161.41	41.25	155.52	84.71	286.80	98.58	251.68	163.29	36.96	158.31	77.96	270.32	104.09	243.15
		b-wave (µV)	267.13	60.14	264.01	155.42	489.81	173.35	387.96	264.59	56.49	261.46	151.83	436.33	169.30	380.20
		*b:a ratio*	*1.69*	*0.27*	*1.66*	*1.15*	*2.76*	*1.23*	*2.32*	*1.64*	*0.26*	*1.63*	*1.10*	*2.68*	*1.18*	*2.28*
	67	a-wave (µV)	188.56	43.77	180.65	101.88	327.04	126.25	294.08	190.24	37.47	185.77	120.58	301.72	131.09	274.69
		b-wave (µV)	276.16	62.71	269.95	151.48	504.27	176.69	391.41	276.16	57.50	272.46	150.38	451.70	172.95	405.85
		*b:a ratio*	*1.48*	*0.22*	*1.47*	*0.90*	*2.32*	*1.09*	*2.00*	*1.46*	*0.21*	*1.46*	*0.86*	*2.17*	*1.08*	*1.92*

The 2.5th and 97.5th percentile are also given to provide a possible reference range, encompassing the central 95% of the data.

ERG amplitudes and b:a ratios deviated from a normal distribution, and so non-parametric testing (paired-sample Wilcoxon Signed Rank Test) was employed for statistical comparisons. Although median b:a ratio for right and left eyes were very similar (as detailed below), there was a statistically significant difference with the ratio being slightly lower in left eyes (p<0.001). For all subsequent analyses, right eyes and left eyes were analysed separately.

**Figure 2:**
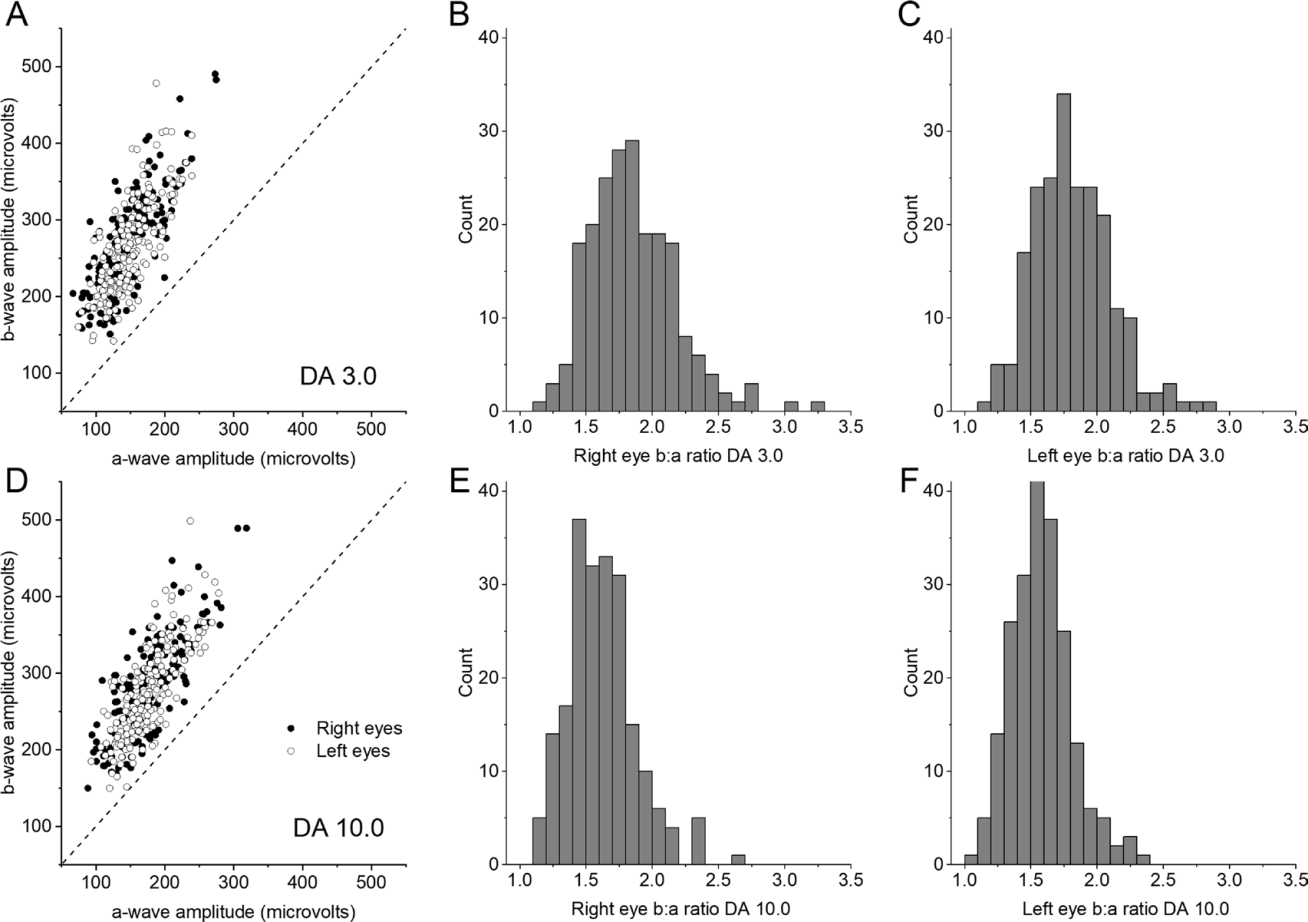
Dark-adapted flash electroretinogram (ERG) amplitudes and b:a ratios for International Society for Clinical Electrophysiology of Vision (ISCEV) standard flashes. Upper panels (A–C) show data for the standard 3.0 cd/m^2^ s flash (termed DA 3 in the ISCEV standard protocol). Lower panels show data for the stronger 10.0 cd/m^2^ s flash of (termed DA 10.0 in the ISCEV standard protocol). Left-hand panels (A, D) plot b-wave amplitudes against a-wave amplitudes for right and left eyes. Electronegative ERGs would be indicated by points falling below the 45° dashed line; no points fall below this line. Right-hand panels (B, C, E, F) are histograms showing the distribution of b:a ratios for right eyes (B, E) and left eyes (C, F). All ratios are greater than 1.

Figure 2 depicts the results: b-wave amplitudes are plotted against a-wave amplitudes in the left-hand panels and the distribution of b:a ratios are shown in the right-hand panels. The distributions suggest that on average b:a ratios were lower for the stronger flash, and this was statistically significant (p<0.0001) though there was considerable overlap. For the standard (DA 3) flash, mean (SD) b:a ratios were 1.86 (0.33) and 1.81 (0.29) for right and left eyes respectively; median (minimum, maximum) values were 1.81 (1.12, 3.25) and 1.79 (1.13, 2.81), respectively. For the stronger (DA 10) flash, mean (SD) b:a ratios were 1.62 (0.25) and 1.58 (0.23) for right and left eyes respectively; median (minimum, maximum) values were 1.60 (1.15, 2.66) and 1.79 (1.05, 2.36), respectively. No recordings from any of the 422 eyes were electronegative. The majority of the participants (99%) were twin pairs. When only one twin from each pair was included (107 unrelated individuals), the same statistically significant relationships were found.

### Additional flash stimuli

**Figure 3:**
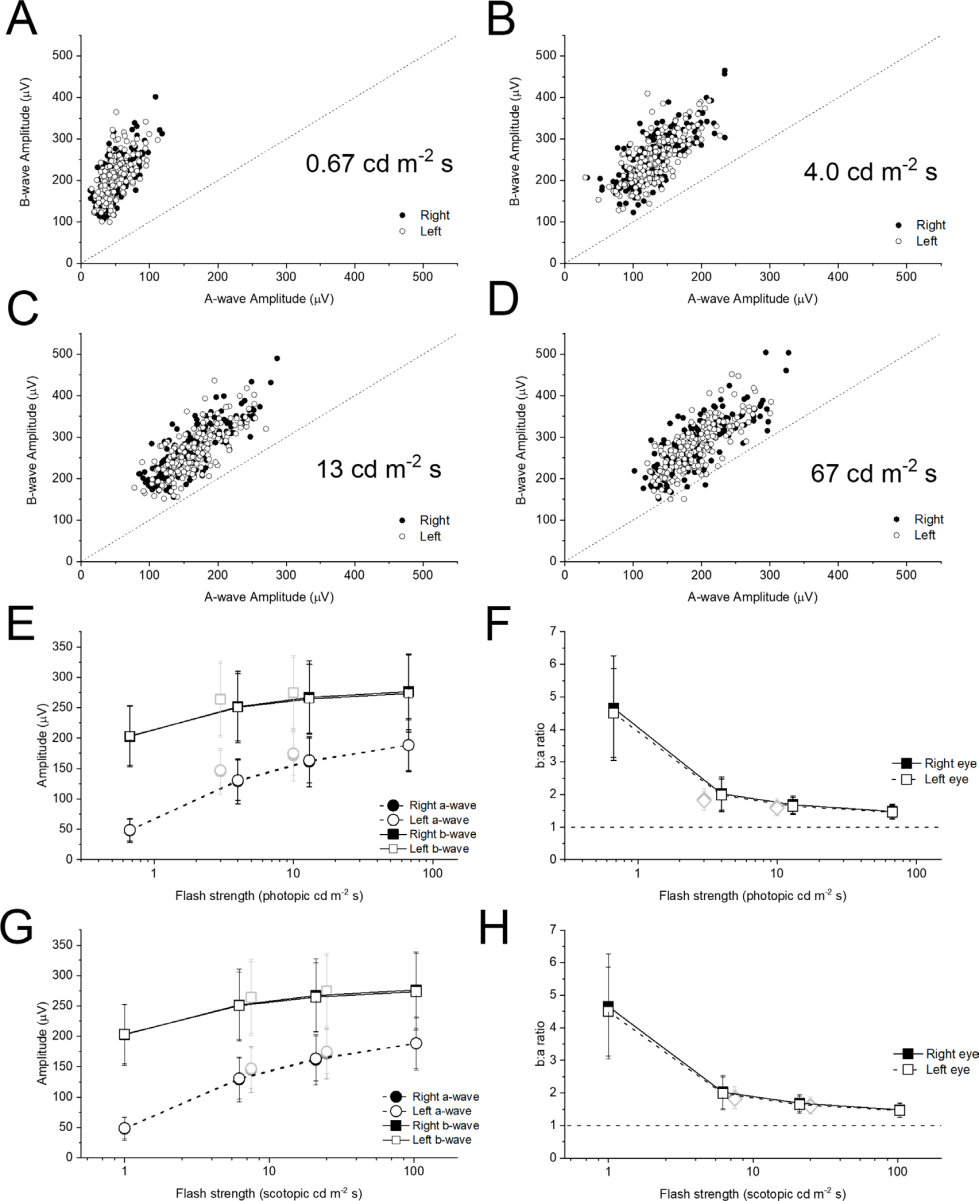
Dark-adapted flash electroretinogram amplitudes and b:a ratios for additional xenon flashes. (A–D) Panels plot b-wave against a-wave amplitudes for the additional xenon flashes (flash strengths shown in photopic units). The scales are consistent to aid comparison. The 45° dashed line indicates a b:a ratio of 1. (E) Points plot mean (±SD) a-wave and b-wave amplitudes for the cohort against flash strength in photopic units. The grey symbols are for the standard LED-derived stimuli (DA 3 and DA 10). (F) Points plot mean (±SD) b:a ratio for the cohort against flash strength. The dashed horizontal line denotes a ratio of unity. (G, H) Plots corresponding to E and F, but where the X-axis represents scotopic flash strength. The photopic flash strengths were measured directly using a photometer with a photopic filter. The scotopic flash strengths are those given by the Espion system for the particular stimuli used.

Figure 3A–D plots b-wave amplitudes against a-wave amplitudes for the additional flash stimuli (all plots are to the same scale). As flash strength increases, the points appear to lie nearer the 45° line, indicating a falling b:a ratio with increasing flash strength, similar to the relationship seen for the ISCEV standard flashes. Again, the difference in b:a ratios between flash strengths was statistically significant, with successively stronger flashes giving significantly lower b:a ratios (p<0.0001). All points are above the 45° line except for the strongest flash: here, 3 points (both eyes of one individual, and the left eye of a second individual) fall below the 45° line. Further inspection of recordings from these participants showed a gradual down-going drift detectable in some traces even prior to flash delivery, suggesting that the b:a ratio was artefactually low. The traces did not however meet the objective criteria for trace rejection.

The lowest panels in figure 3 plot mean (±SD) a-wave and b-wave amplitudes (lower left panels) and b:a ratio (lower right panels) against flash strength for the cohort. In panels E and F, the X-axis represents photopic flash strengths (stimuli are conventionally given in photopic units). However, as the responses are largely from the rod system, the scotopic strength may be more relevant. In panels G and H, the X-axis represents scotopic strengths (as given by the Espion system for the stimuli used). The grey symbols represent the ISCEV standard (DA 3 and DA 10) stimuli. The scotopic strength of a given white LED stimulus is higher than that of a photopically matched xenon flash (reflecting a greater contribution of shorter (bluer) wavelengths in the LED spectrum). As the dark-adapted responses are largely rod driven, this is likely to explain why the grey symbols appear to be more consistent with the black symbols when flash strength is plotted in scotopic, rather than photopic, units.

## Discussion

In this study, we investigated recordings obtained previously from 211 adult participants from the TwinsUK cohort (with ages ranging from 23 to 86 years) who underwent ISCEV standard recording of ERGs. The primary aim at the time of the recordings had been to investigate heritability of response parameters and investigate factors such as correlation with age^12^; no recordings were excluded on the basis of waveform shape or relative amplitudes of waveform components. We found that, in response to international standard stimuli, no participant had an electronegative waveform. For additional stimuli, we found the b:a ratio became smaller as flash strength increased, but was still greater than 1. For the strongest flash (67 photopic cd/m^2^ s), b:a ratios less than 1 were only observed in three eyes, and this was equivocal (with the possibility that the ratio was artefactually low in these recordings).

There was a small, but statistically significant, difference in b:a ratio between right and left eyes. It is unclear whether this reflects a true interocular difference or might simply arise from differences in relative electrode position, for example, resulting in opposite directions of mild drift in the signal that might be minimally discernible, but could lead to a statistically significant difference in a large sample. The median values for right and left eyes were close to each other.

The reduction in b:a ratio with increasing flash strength is well established,^16 17^ and confirmed in our study, suggesting an earlier saturation of b-wave amplitudes relative to a-wave amplitudes. The particular stimulus strength at which response amplitudes are deemed to be maximal can depend on the model used to fit the relationship.^18^ A recently published ISCEV extended protocol for the stimulus–response series for the dark-adapted ERG b-wave summarises previous approaches.^19^ With hyperbolic saturation functions commonly used, the maximal amplitude is often fitted to a plateau that is reached with weaker flash strengths than those used here. The flash strengths of the current study probably correspond to the ‘second limb’ of the stimulus–response relation, in which b-wave amplitudes continue to increase following that plateau.^19^

There is significant amplification at the rod to rod-bipolar cell synapse, with bipolar cells integrating input from multiple rod photoreceptors, and this might lead to saturation in bipolar cell response amplitude at lower stimulus strengths than for the rod photoreceptors themselves. Additionally, it has been shown that the a-wave trough in response to strong flashes is likely to be shaped not just by the outer segment photocurrent, but by current flows elsewhere including the outer nuclear layer,^20^ and this additional component might continue to increase with flash strength. Although dominated by the rod system, the dark-adapted ERG elicited by a strong flash additionally contains signals from the cone system (cone photoreceptors, ON and OFF bipolar cells), which could also affect the b:a ratio.

Although our sample is large, and covers a substantial age range, we cannot exclude that an electronegative waveform might still occur in a very small proportion (less than 1%) of healthy subjects. Our cohort has specific demographics, and we cannot be certain of how our findings would generalise to other populations. The predominance of females is partly explained by the original founding aim of the twin registry, which was to investigate osteoporosis and osteoarthritis, conditions more prevalent in women.^13^ Our findings only apply to the strengths analysed (which include the ISCEV standards). Electronegative waveforms can be a normal finding in some stimulus conditions, and the b:a ratio can be lower with stronger flashes (as confirmed by our study). Some experimental testing protocols might employ significantly stronger flashes than the those analysed here, and our findings would not apply to these flash strengths.

Also, manual rejection of artefactual traces can be subjective, but we adhered to the criteria described in the Materials and methods. It is possible that different strategies for rejection of traces (eg, varying levels of tolerance to drift in the recording) might affect the prevalence of electronegative waveforms in some populations. However, in our study, the appearance of an electronegative response was not a criterion for trace rejection. The few electronegative ERGs seen in response to our strongest stimulus were felt to be possibly related to drift in the recording that did not exceed our threshold for trace rejection. It is possible that recording over a larger time window would have enabled more robust identification of sustained drift in the recordings.

Dark-adapted electronegative waveforms can be an important diagnostic finding, which can guide genetic testing, or be indicative of significant systemic pathology (potentially life-threatening, as in cases of paraneoplastic retinopathy).^21^ Our findings support the notion that, when observed, electronegative ERGs should be regarded as clinically significant, and, when unexpected, should prompt further exploration, by medical history, and/or ocular or systemic investigations as appropriate.

## Data Availability

Data are available upon request.

## References

[1] Robson AG, Nilsson J, Li S, et al. ISCEV guide to visual electrodiagnostic procedures. Doc Ophthalmol 2018;136:1–26. 10.1007/s10633-017-9621-y PMC5811581 29397523

[2] McCulloch DL, Marmor MF, Brigell MG, et al. ISCEV standard for full-field clinical electroretinography (2015 update). Doc Ophthalmol 2015;130:1–12. Erratum in: Doc Ophthalmol. 2015 Aug;131(1):81-3. 10.1007/s10633-014-9473-7 25502644

[3] Jiang X, Mahroo OA. Negative electroretinograms: genetic and acquired causes, diagnostic approaches and physiological insights. Eye 2021. 10.1038/s41433-021-01604-z. [Epub ahead of print: 14 Jun 2021]. PMC8377097 34127841

[4] Audo I, Robson AG, Holder GE, et al. The negative ERG: clinical phenotypes and disease mechanisms of inner retinal dysfunction. Surv Ophthalmol 2008;53:16–40. 10.1016/j.survophthal.2007.10.010 18191655

[5] Robson AG, Richardson EC, Koh AHC, et al. Unilateral electronegative ERG of non-vascular aetiology. Br J Ophthalmol 2005;89:1620–6. 10.1136/bjo.2005.071357 16299143 PMC1772970

[6] Koh AH, Hogg CR, Holder GE. The incidence of negative ERG in clinical practice. Doc Ophthalmol 2001;102:19–30. 10.1023/A:1017586118749 11475363

[7] Renner AB, Kellner U, Cropp E, et al. Dysfunction of transmission in the inner retina: incidence and clinical causes of negative electroretinogram. Graefes Arch Clin Exp Ophthalmol 2006;244:1467–73. 10.1007/s00417-006-0319-1 16612636

[8] Kim JM, Payne JF, Yan J, et al. Negative electroretinograms in the pediatric and adult population. Doc Ophthalmol 2012;124:41–8. 10.1007/s10633-011-9301-2 22246197

[9] Alsalamah AK, Khan AO. Electronegative electroretinograms in the United Arab Emirates. Middle East Afr J Ophthalmol 2020;27:86–90. 10.4103/meajo.MEAJO_106_20 32874040 PMC7442078

[10] Dhingra A, Fina ME, Neinstein A, et al. Autoantibodies in melanoma-associated retinopathy target TRPM1 cation channels of retinal ON bipolar cells. J Neurosci 2011;31:3962–7. 10.1523/JNEUROSCI.6007-10.2011 21411639 PMC3073846

[11] Kondo M, Sanuki R, Ueno S, et al. Identification of autoantibodies against TRPM1 in patients with paraneoplastic retinopathy associated with ON bipolar cell dysfunction. PLoS One 2011;6:e19911. 10.1371/journal.pone.0019911 21611200 PMC3096646

[12] Bhatti T, Tariq A, Shen T, et al. Relative genetic and environmental contributions to variations in human retinal electrical responses quantified in a twin study. Ophthalmology 2017;124:1175–85. 10.1016/j.ophtha.2017.03.017 28434717 PMC5540060

[13] Verdi S, Abbasian G, Bowyer RCE, et al. Twinsuk: the UK adult twin registry update. Twin Res Hum Genet 2019;22:523–9. 10.1017/thg.2019.65 31526404

[14] Kurtenbach A, Kramer S, Strasser T, et al. The importance of electrode position in visual electrophysiology. Doc Ophthalmol 2017;134:129–34. 10.1007/s10633-017-9579-9 28224239

[15] Paupoo AA, Mahroo OA, Friedburg C, et al. Human cone photoreceptor responses measured by the electroretinogram [correction of electoretinogram] a-wave during and after exposure to intense illumination. J Physiol 2000;529(Pt 2):469–82. 10.1111/j.1469-7793.2000.00469.x 11101655 PMC2270196

[16] Breton ME, Schueller AW, Montzka DP. Electroretinogram b-wave implicit time and b/a wave ratio as a function of intensity in central retinal vein occlusion. Ophthalmology 1991;98:1845–53. 10.1016/S0161-6420(91)32057-8 1723186

[17] Freund PR, Watson J, Gilmour GS, et al. Differential changes in retina function with normal aging in humans. Doc Ophthalmol 2011;122:177–90. 10.1007/s10633-011-9273-2 21562738

[18] Severns ML, Johnson MA. The care and fitting of Naka-Rushton functions to electroretinographic intensity-response data. Doc Ophthalmol 1993;85:135–50. 10.1007/BF01371129 7521824

[19] Johnson MA, Jeffrey BG, Messias AMV, et al. ISCEV extended protocol for the stimulus-response series for the dark-adapted full-field ERG b-wave. Doc Ophthalmol 2019;138:217–27. 10.1007/s10633-019-09687-6 30929109 PMC11332362

[20] Robson JG, Frishman LJ. The rod-driven a-wave of the dark-adapted mammalian electroretinogram. Prog Retin Eye Res 2014;39:1–22. 10.1016/j.preteyeres.2013.12.003 24355774 PMC3939025

[21] Roels D, Ueno S, Talianu CD, et al. Unilateral cancer-associated retinopathy: diagnosis, serology and treatment. Doc Ophthalmol 2017;135:233–40. 10.1007/s10633-017-9605-y 28815346

